# Notes and News

**Published:** 1906-02-17

**Authors:** 


### NOTES AND NEWS.

LORD MONKSWELL will preside at the annual general meeting of University College Hospital on Thursday, March 22.

THE Pharmacy Acts Amendment Bill, which has been before the House of Commons during many recent sessions, is to be introduced in the new Parliament at an early date, and it is stated that about half the members have expressed themselves in agreement with its principles.

THE Battersea Borough Council have recently had under consideration a proposal for the establishment of voluntary notification of tuberculosis as an infectious disease. They have now decided definitely not to adopt any scheme which includes the payment of medical practitioners for their certificates of notification.

AT the last meeting of the Lewisham Guardians the salary of Dr. Toogood, resident medical officer at Lewisham Infirmary, was increased from £325 to £400. One of the Guardians declared that he was "worth his weight in gold," and that through his knowledge cures of cases had been effected which would otherwise have been hopeless.

THE Automobile Mutual Protection Association and the Roads Improvement Association are taking active steps to prove that dustless roads can be constructed which will involve less cost to the ratepayers than the present dusty roads. Arrangements are being made with the Kent County Council to experiment on a section of the London and Maidstone road.

IN the course of last year no fewer than 475 cases of alleged infringement of the Pharmacy Acts were investigated by the Registrar of the Pharmaceutical Society. The alleged offences included the sale of poisons by unqualified persons, the assumption of the title of chemist by such unqualified persons, and neglect of proper precautions in labelling poisons and using distinctive bottles.

ABOUT 40 persons have been ill through eating potted meat bought at a co-operative store in Accrington, and there has been one death from the same cause.

THE Royal Commission on Tuberculosis will shortly issue a detailed report upon the results of the experimental investigations which have been conducted on two farms at Stansted, in Essex.

PROFESSOR RUBERT BOYCE, F.R.S., has kindly consented to give an address at the forthcoming International Medical Congress on "The Prophylaxes of Yellow Fever as the result of the 1905 Epidemic in Central America and New Orleans." This address will take the place of the communication promised by Sir Patrick Marian, K.C.M.G., who is unavoidably prevented from attending the meeting at Lisbon.

AT the annual meeting of the Royal London Ophthalmic Hospital Guild the Chairman of the Hospital Committee, who presided, stated that the Guild supplied all the linen and clothes required by the great institution in the City Road. It also endowed a bed and a cot in the hospital, and during the last two years it had made very handsome donations to the Centenary Fund, founded to provide for the heavy rental of £1,200.

SIR LAUDER BRUNTON, speaking at Bristol this week at a meeting under the auspices of the National League for Physical Education, referred to the great waste of human life through ignorance, carelessness, or injurious sanitary surroundings, and said that it was time the dreadful destruction of infant life was prevented. He believed that a great deal would be done by a general system of physical training, which he advocated as a national obligation.

AN appeal is made by Canon Newbolt and the authorities of the Guild of St. Luke for funds in order to meet the need of supplying well qualified medical men in the mission field. Ever since there has been a medical branch of the missionary service the demand for men has been vastly in excess of the supply, and the Guild of St. Luke are anxious to be able to add to the two students now in training at the residential college of the institution on Carlton Hill, N.W., which was recently opened by the Bishop of London.

MR. BENJAMIN ISAAC, of 11 Albert Hall Mansions, W., and of Great Winchester Street, E.C., who died last December, bequeathed £500 to the Jews' Hospital and Orphan, £500 to the London Hospital, £300 to the Royal Free Hospital, £200 each to the Cancer Hospital (Brompton), the Hospital for Consumption at Brompton, the New Hospital for Women, St. Mary's Hospital (Paddington), the Great Northern Hospital, and the Metropolitan Hospital, as well as £100 each to Guy's Hospital, St. Thomas's Hospital, and several other London institutions.

